# SG-APSIC1132: BHQ isolation cart management at a Bangkok Hospital

**DOI:** 10.1017/ash.2023.112

**Published:** 2023-03-16

**Authors:** Rome Chomrak

**Affiliations:** Bangkok Hospital, Bankok, Thailand

## Abstract

**Objectives:** We aimed to provide sufficient equipment to effectively and efficiently track all equipment. Advanced management procedures are available for the treatment of any patients with infections or immunodeficiency as well as those who need special treatment or isolation from others. This management plan can be beneficial for the hospital by promoting process improvement and achieving cost effectiveness. **Methods:** The instruments used were surveyed in all departments of Bangkok Hospital and these data were analyzed. We used a plan–do–check–act (PDCA) strategy to improve the management by moving from a manual system to a collaborative innovation project that used 4 technological systems: (1) storage identification and identification codes for equipment; (2) request, return, and delivery using the Nsmart system; (3) transportation, receiving, and delivery; and (4) an HIS system for tracing and NSterile version 4.0 software for reporting. **Results:** The BHQ isolation cart management system helped the hospital control inventory and prevent infection and helped standardize patient services to improve quality at the hospital. **Conclusions:** This report confirms earlier findings that sufficient equipment can be made available to patients with no extra cost to management. Our findings can contribute to efforts to prevent the virus from spreading within the hospital.

